# Hydrogen sulfide as a hypoxia mimetic for improving chronic neural implant integration

**DOI:** 10.3389/fnins.2026.1844345

**Published:** 2026-09-23

**Authors:** Ladan Jiracek-Sapieha

**Affiliations:** Department of Electrical and Computer Engineering, University of Florida, Gainesville, FL, United States

**Keywords:** gasotransmitters, gliosis, hydrogen sulfide, hypoxia-inducible factor-1α, implant integration, neural implants, neuroinflammation, reactive oxygen species

## Abstract

Chronic neural implants often exhibit progressive loss of function due to a foreign body response characterized by inflammation, oxidative stress, and glial scarring at the implant-tissue interface. While advances in device mechanics and materials have improved performance, complementary bioactive strategies are needed to modulate the local biological environment and enhance long-term stability. Here, we propose that hydrogen sulfide (H₂S), an endogenously produced gasotransmitter with cytoprotective, antioxidant, and neuromodulatory functions, can be leveraged as a localized hypoxia mimetic at the neural implant interface. Specifically, we advance the hypothesis that controlled, low-dose H₂S delivery can transiently engage hypoxia-responsive signaling pathways, including modulation of mitochondrial respiration, stabilization of hypoxia-inducible factor-1α, and activation of redox-sensitive transcriptional programs, to shift the foreign body response toward a more permissive, pro-survival state. This framework integrates mechanistic insights from H₂S biology with the physiological constraints of the peri-implant microenvironment, emphasizing spatial confinement, temporal control, and dose precision as key design parameters. We examine how these constraints shape potential delivery strategies and highlight the limitations of passive release approaches for achieving consistent, therapeutically relevant signaling. We further outline testable predictions and experimental pathways for evaluating this hypothesis, alongside key translational challenges including dose regulation, *in situ* sensing, and regulatory classification. Together, this work defines a testable conceptual framework for modulating the neural implant-tissue interface through controlled biochemical signaling and positions H₂S-based strategies as a promising, but tightly constrained, direction for improving the performance and longevity of implantable neurotechnologies.

## 1 Introduction

Implantable neural devices, including intracortical electrodes and neurostimulation systems, have enabled new therapies and brain-machine interfaces, but their long-term performance is often undermined by the foreign body response. Implantation triggers inflammation, oxidative stress, and glial scar formation around the device, leading to neuronal loss, electrical isolation, and progressive signal degradation (Polikov et al., 2005). These processes are especially dynamic during the acute-to-subacute post-implantation period (days to weeks), when inflammatory and metabolic responses are most biologically tractable (Polikov et al., 2005).

To address these challenges, bioactive strategies are increasingly explored alongside advances in device materials and mechanics. One promising avenue involves endogenous gasotransmitters, such as nitric oxide (NO), carbon monoxide (CO), and hydrogen sulfide (H₂S), which regulate inflammation, redox balance, and tissue repair (Mustafa et al., 2009). The discovery of NO as a biological signaling molecule was recognized with the 1998 Nobel Prize in Physiology or Medicine, underscoring the physiological relevance of gasotransmitter signaling in complex tissues (Nobel Prize Outreach, 1998). Hydrogen sulfide, once regarded primarily as a toxic gas, is now recognized as an endogenous neuromodulator with cytoprotective, anti-inflammatory, and pro-angiogenic effects in the nervous system (Abe and Kimura, 1996; Kimura et al., 2012). H₂S also interacts with NO signaling and modulates mitochondrial metabolism and redox-sensitive pathways (Mustafa et al., 2009), positioning it as a candidate for mitigating implant-induced tissue responses. Importantly, many of the protective mechanisms engaged by H₂S overlap with canonical hypoxia-responsive signaling programs.

Implantation further disrupts local vasculature, increases metabolic demand, and introduces cellular infiltration at the device interface, creating conditions of localized mismatch between oxygen supply and consumption (Kozai et al., 2014). While not necessarily constituting classical hypoxia, this microenvironment shares key features of hypoxic stress, including elevated oxidative load and activation of stress-responsive signaling pathways. Testing this concept will require physiologically relevant animal implantation models capable of capturing the coupled vascular, inflammatory, electrophysiological, and device-level responses that cannot be reproduced fully *in vitro* (Choudhary, 2025). Emerging artificial intelligence and machine-learning approaches may further assist in integrating multidimensional electrochemical, electrophysiological, and biological measurements to characterize dose–response relationships and, ultimately, support adaptive control of localized H₂S delivery (Cernat et al., 2024).

Accordingly, this Hypothesis and Theory article advances the testable hypothesis that spatially confined, temporally bounded H₂S delivery during the acute-to-subacute response to neural implantation can improve chronic neural interface integration by transiently engaging hypoxia-linked, antioxidant, and anti-inflammatory pathways without inducing sustained pseudohypoxia or sulfide toxicity.

### 1.1 Central hypothesis

Here, we propose that localized, low-dose hydrogen sulfide (H₂S) delivery at the neural implant-tissue interface can act as a controlled hypoxia mimetic, inducing a transient, spatially confined pseudohypoxic state that shifts the foreign body response from a pro-inflammatory, oxidative phenotype toward reduced inflammatory signaling, greater neuronal preservation, and improved tissue–device integration. This effect is hypothesized to arise through coordinated modulation of mitochondrial respiration, stabilization of hypoxia-inducible factor-1α (HIF-1α), and activation of redox-sensitive cytoprotective pathways (e.g., Nrf2), collectively promoting neuronal preservation and improved device integration.

This framework differs from prior work in two key respects. First, it does not seek to comprehensively review H₂S biology, which has been extensively covered elsewhere (Paul and Snyder, 2018; Morris et al., 2025), but instead focuses on a specific, testable application at the neural interface. Second, it emphasizes spatial confinement, temporal control, and interface-specific biology—design constraints that fundamentally distinguish implant-based delivery from systemic H₂S therapies developed for neurological disease. Accordingly, H₂S is considered here not as a general neuroprotective agent, but as a locally tunable modulator of the device-tissue microenvironment.

## 2 Mechanistic basis: H₂S signaling in the neural Interface context

Hydrogen sulfide is endogenously produced in mammalian tissues, including the nervous system, by cystathionine β-synthase (CBS), cystathionine γ-lyase (CSE), and 3-mercaptopyruvate sulfurtransferase (3-MST) (Kimura et al., 2012; Paul and Snyder, 2015). In the brain, CBS, primarily expressed in astrocytes but also present in neurons, is a major source of H₂S, while CSE predominates in peripheral tissues. Mitochondrial 3-MST can also generate H₂S in concert with cysteine aminotransferase. H₂S has been shown to modulate synaptic activity, including NMDA receptor function, establishing its role as an endogenous neuromodulator in the central nervous system (Abe and Kimura, 1996; Kimura, 2000; Kimura, 2011). For neural interface applications, this endogenous signaling capacity indicates that neural tissue is already equipped to respond to sulfide-based cues within a physiological concentration range.

Beyond neuromodulation, H₂S exerts cytoprotective effects in neural cells through redox and mitochondrial mechanisms. H₂S enhances antioxidant defenses, including glutathione synthesis, and activates the nuclear factor erythroid 2-related factor 2 (Nrf2) pathway, thereby limiting reactive oxygen species (ROS)-mediated damage (Kimura et al., 2012; Shefa et al., 2017). It also engages pro-survival signaling pathways such as protein kinase B (Akt) and extracellular signal-regulated kinase (ERK) (Shefa et al., 2017) and can transiently inhibit mitochondrial respiration at higher concentrations, reducing oxidative stress burden (Szabo, 2018). In the context of neural implants, these mechanisms are directly relevant to the peri-implant environment, where oxidative stress and metabolic disruption contribute to neuronal loss and glial activation.

H₂S additionally intersects with injury-relevant pathways that shape inflammation and repair. In neuroinjury models, H₂S donors reduce apoptotic signaling and oxidative stress (Wei et al., 2014). H₂S is also an endogenous stimulator of angiogenesis (Papapetropoulos et al., 2009), which may be relevant to vascular remodeling near chronically implanted devices. H₂S has also been implicated in peripheral nerve degeneration and regeneration processes (Jung and Jeong, 2014; Wang et al., 2012). Collectively, these findings support the premise that H₂S signaling can influence oxidative stress, inflammatory tone, and tissue remodeling—key determinants of chronic neural implant performance.

Notably, several effects of H₂S parallel those of hypoxic or ischemic preconditioning, in which brief hypoxia induces resistance to subsequent stress. Both H₂S and hypoxia activate ATP-sensitive K^+^ (K_ATP) channels and stress-responsive gene programs, supporting the concept that H₂S can function as a hypoxia mimetic in triggering cellular defense mechanisms (Tan et al., 2010).

## 3 H₂S as a hypoxia mimetic

Intermittent or time-limited hypoxic exposure has been shown to induce neuroprotective plasticity in the central nervous system, including enhancement of synaptic function, neurotrophic signaling, and functional recovery following injury (Golder and Mitchell, 2005; Hayes et al., 2014; Navarrete-Opazo and Mitchell, 2014). These adaptive responses are mediated in part through hypoxia-inducible signaling pathways that promote cellular resilience under stress. One of the most compelling aspects of H₂S biology is its overlap with these hypoxia-responsive signaling pathways. Under low-oxygen conditions, cells stabilize hypoxia-inducible factor-1α (HIF-1α), which induces gene programs that support adaptation and survival. Exogenous H₂S can activate similar responses even under normoxic conditions. In *C. elegans*, H₂S exposure stabilizes HIF-1 and induces hypoxia-responsive genes, while loss of HIF-1 abolishes H₂S-mediated survival benefits (Miller and Roth, 2007). In mammalian systems, H₂S similarly stabilizes HIF-1α and increases expression of downstream targets such as vascular endothelial growth factor (VEGF) in the absence of true hypoxia (Zhang et al., 2017; Huang et al., 1996). These effects are thought to arise from inhibition of prolyl hydroxylase (PHD) enzymes, via redox modulation or interaction with their iron cofactors, thereby preventing HIF-1α degradation. In parallel, H₂S partially and reversibly inhibits mitochondrial cytochrome c oxidase (complex IV), reducing oxygen consumption and ROS generation and reinforcing HIF-1α stabilization under normoxia (Blackstone et al., 2005).

H₂S also activates the Nrf2 antioxidant response and modulates mitochondrial respiration, further aligning its signaling profile with hypoxic adaptation (Hine et al., 2015). Hypoxia-responsive pathways are strongly dependent on dose, timing, and exposure pattern: transient activation promotes adaptive plasticity, whereas sustained activation can be ineffective or harmful (Navarrete-Opazo and Mitchell, 2014). This constraint is central to implant-based H₂S strategies, as prolonged activation may shift signaling from protective to maladaptive. This principle underlies intermittent hypoxia therapies, where brief exposure induces durable functional benefits without the pathological consequences of continuous hypoxia (Golder and Mitchell, 2005; Hayes et al., 2014). A key advantage of H₂S is its potential to engage these pathways locally at an implant site without systemic oxygen deprivation.

At higher concentrations, H₂S can induce profound metabolic suppression. In mice, inhaled H₂S has been reported to reduce oxygen consumption and core temperature, mimicking a suspended-animation-like state (Blackstone et al., 2005; Morrison et al., 2008), though this effect has not been consistently reproduced (Hemelrijk et al., 2018). These divergent findings underscore the context-, species-, and dose-dependence of H₂S-mediated hypoxia-mimetic effects and limit direct extrapolation to localized, device-mediated delivery.

H₂S does not fully replicate hypoxia, but instead selectively engages components of the hypoxic response under normoxic conditions. This partial activation may be advantageous, but introduces uncertainty in pathway specificity, magnitude, and duration. Sustained HIF-1α stabilization, for example, has been linked to angiogenesis and metabolic reprogramming, which may be beneficial acutely but maladaptive if chronically maintained.

In the context of neural implants, localized H₂S delivery could harness hypoxia-linked protective programs at the device-tissue interface. By stabilizing HIF-1α and related adaptive pathways, H₂S may promote angiogenesis and antioxidant defense while reducing oxidative and inflammatory stress at the implant interface; its direct anti-inflammatory actions may further attenuate chronic gliosis. Critically, the objective is not sustained pseudohypoxia, but transient, spatially confined modulation during the acute-to-subacute post-implantation window, when tissue responses are most plastic. A consolidated view of these mechanisms is shown in Figure 1.

**Figure 1:**
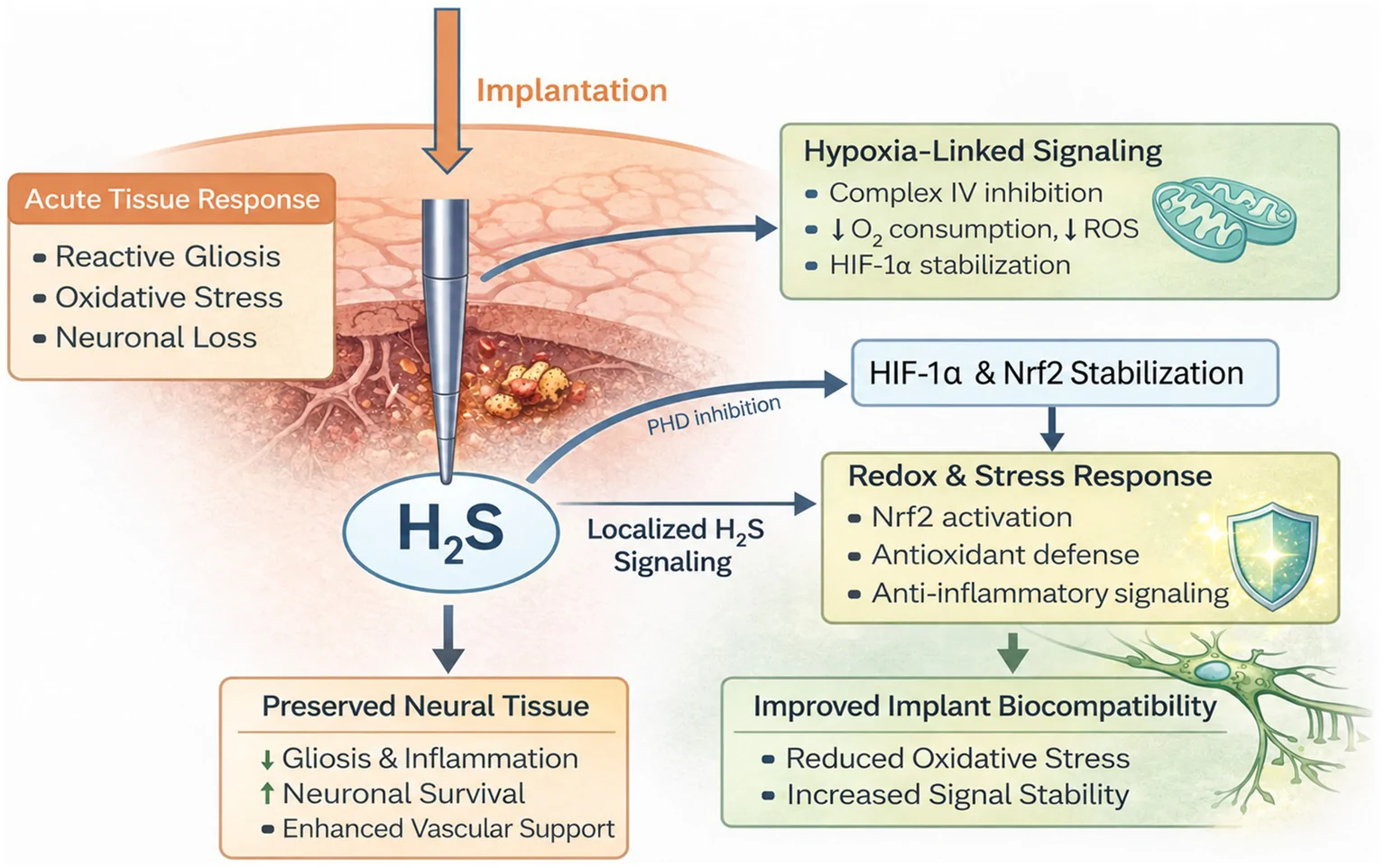
Mechanistic overview of proposed H₂S-mediated modulation at the neural implant-tissue interface. Localized H₂S signaling is hypothesized to reduce oxidative stress, gliosis, and neuronal loss through combined effects on cytochrome c oxidase, prolyl hydroxylase activity, HIF-1α stabilization, and Nrf2-linked antioxidant signaling.

## 4 Preclinical evidence of H₂S in neuroprotection

Preclinical studies across models of acute neural injury and chronic neurodegeneration demonstrate that exogenous hydrogen sulfide can preserve neural tissue and function, supporting its potential relevance to neural implant integration (Zhang et al., 2017; Li et al., 2017). In spinal cord injury (SCI) models, H₂S has shown particularly compelling protective effects. Administration of H₂S donors in rodent models of spinal cord ischemia–reperfusion injury reduces neuronal cell death, limits tissue damage, and improves locomotor recovery (Li et al., 2015). Mechanistic analyses indicate that these benefits are associated with enhanced autophagy, suppression of apoptotic signaling, preservation of mitochondrial integrity, and attenuation of oxidative stress within the injured spinal cord.

Across Alzheimer’s, Parkinson’s, neuroinflammatory, and peripheral nerve injury models, H₂S consistently reduces oxidative stress, inflammatory signaling, and neuronal injury (Wei et al., 2014; Jung and Jeong, 2014; Giuliani et al., 2013; Giovinazzo et al., 2021; Hu et al., 2010; Xue and Bian, 2015). These findings primarily arise from systemic or bolus delivery paradigms that differ substantially from the localized, interface-specific conditions of neural implants. Accordingly, the magnitude, spatial distribution, and temporal dynamics of H₂S exposure at an implant site may differ significantly from those observed in these models.

Despite these differences, the observed effects converge on processes central to implant-induced tissue failure, including oxidative stress, mitochondrial dysfunction, inflammatory signaling, and neuronal loss. These processes closely mirror the oxidative and inflammatory cascades observed at the neural implant interface, further supporting their relevance to device-induced tissue response. This convergence provides mechanistic justification—but not direct validation—for applying H₂S-based strategies to neural interfaces. Notably, several studies report durable functional benefits following transient H₂S exposure, suggesting that brief pathway engagement may yield lasting effects. Collectively, these findings support H₂S as a neuroprotective modulator and provide a mechanistic basis for exploring its use as a localized, time-limited intervention at the neural implant-tissue interface.

## 5 Opportunities in neural implants

Coupling the biological effects of H₂S with neurotechnology opens new possibilities to enhance the longevity and performance of neural implants. Localized, low-dose H₂S delivery at the implant-tissue interface could dampen oxidative stress and inflammation during the early post-implantation period, when tissue responses are most plastic. Flexible intracortical electrodes and next-generation neuromodulation systems could, in principle, be augmented by transient biochemical modulation to support improved neuronal preservation and reduced peri-implant inflammation. The goal is transient engagement of protective pathways rather than continuous modulation. This approach aligns with the broader objectives of bioelectronic medicine, which seeks to integrate biological and electronic therapies.

However, the feasibility of implementing controlled H₂S delivery in neural implants is constrained by competing requirements for dose precision, spatial localization, device compatibility, and long-term stability. These constraints fundamentally distinguish neural interfaces from conventional drug delivery platforms and limit the direct translation of existing H₂S delivery strategies.

Several strategies, summarized in Figure 2, are proposed to integrate H₂S delivery with neural implants, including both passive release systems and emerging approaches that enable active, electrically controlled delivery:

1. *Injectable slow-release donors*: Periodic systemic or peri-implant administration of slow-releasing donors (e.g., GYY4137) allows adjustable dosing and is well suited for proof-of-concept studies. Slow-release donor systems provide a useful platform for sustained sulfide exposure, although implant-specific exposure profiles would need to be determined experimentally (Lee et al., 2011). However, this strategy lacks spatial precision at the microscale of the implant interface and may produce heterogeneous exposure profiles that are difficult to control experimentally.
2. *Hydrogel or polymer coatings*: Biocompatible coatings loaded with H₂S donors could, in principle, provide localized diffusion-based delivery from the implant surface. In addition to passive diffusion, such coatings could potentially be adapted to incorporate electro-activatable precursor systems, enabling externally or device-triggered sulfide release. Preliminary work controlling H₂S exposure in PDMS-based systems supports the broader feasibility of diffusion-mediated sulfide control, although this remains far from a validated chronic neural implant strategy (Christoforidis et al., 2017). However, donor stability, long-term H₂S retention, and mechanical compatibility with probe insertion present substantial challenges, and such approaches may be better suited to short-term experimental use than chronic implantation.
3. *Nanoparticles or microspheres*: Peri-implant depots of H₂S-releasing particles can extend local exposure without direct integration into the device (Do et al., 2019). In principle, functionalized particles could also be engineered to respond to electrical or electrochemical cues, although such approaches remain highly exploratory. Rapid clearance, spatial dispersion, and regulatory complexity may limit their effectiveness for sustained modulation, favoring transient or adjunctive use. These limitations are particularly relevant in the central nervous system, where immune clearance and tissue diffusion can rapidly dissipate localized delivery.

**Figure 2:**
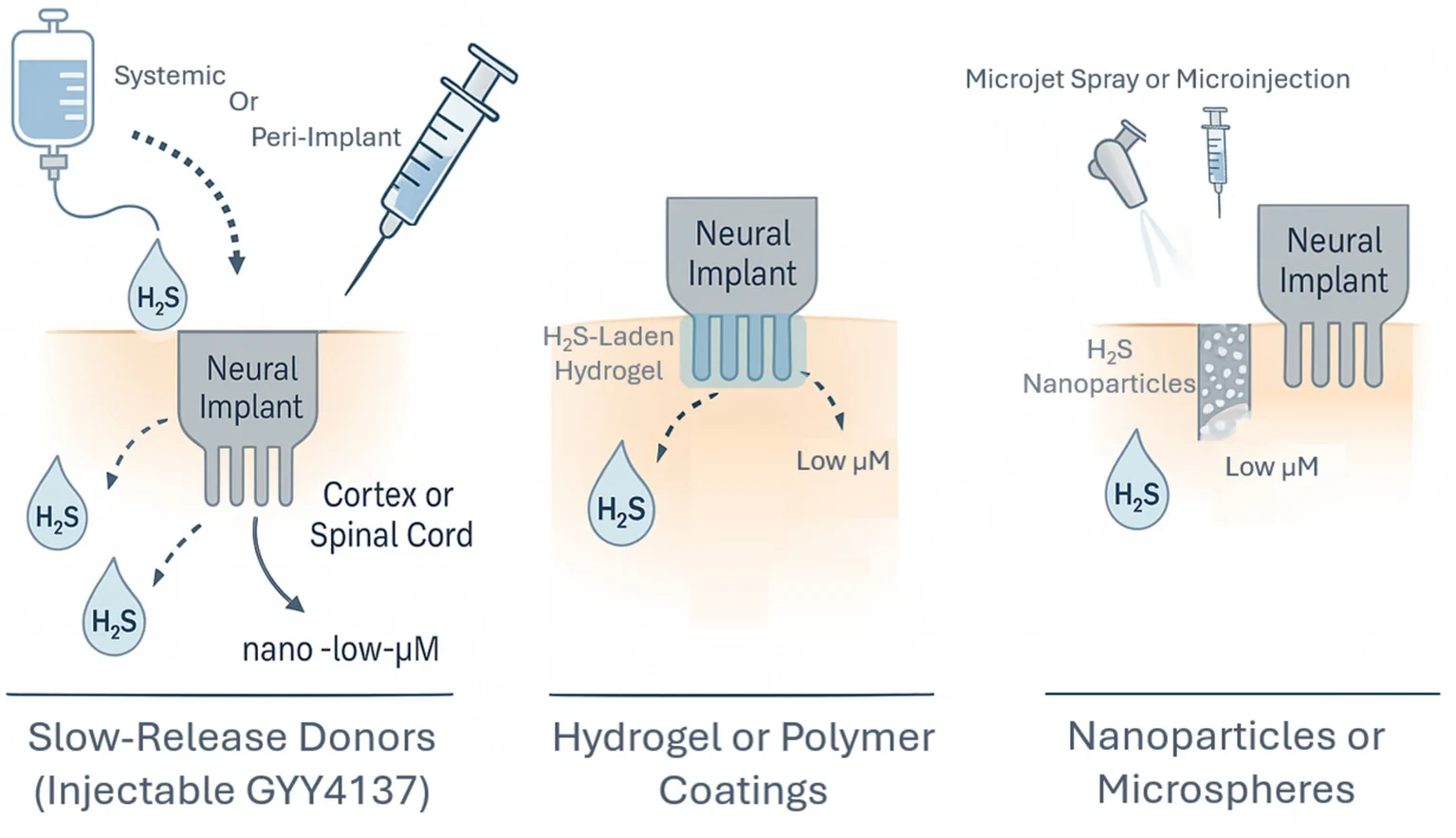
H₂S Delivery Strategies for Neural Implants. Schematic overview of three approaches for delivering hydrogen sulfide (H₂S) to neural implant sites: (1) Slow-Release Donors (Injectable): Periodic administration of slow-releasing H₂S donor compounds (e.g., GYY4137) as a proof-of-concept strategy for sustained sulfide exposure (Lee et al., 2011). (2) Hydrogel or Polymer Coatings: Application of an H₂S-laden hydrogel or polymer coating to the implant as a conceptual route for localized diffusion-based delivery, with only preliminary precedent for controlled H₂S exposure in related PDMS-based systems (Christoforidis et al., 2017). (3) Nanoparticles or microspheres deployed peri-implant as transient depots (Do et al., 2019). Each strategy presents distinct advantages and constraints in terms of dose control, duration, and translational feasibility. All strategies may also be compatible with electro-activated H₂S precursor delivery, in which in-built electrodes generate brief, localized H₂S release on demand. Concentration labels indicate approximate exposure regimes reported or commonly examined in existing H₂S delivery studies and are provided for conceptual illustration only; an implant-specific therapeutic concentration range has not yet been established.

A central unresolved issue across all three delivery strategies is the relationship between nominal donor loading, actual peri-implant H₂S concentration, and biological response. Existing studies provide platform-specific measurements of release kinetics and sulfide exposure (Lee et al., 2011; Christoforidis et al., 2017; Do et al., 2019), but these values cannot yet be interpreted as an established therapeutic range for neural implants. Local concentration will depend on donor chemistry, diffusion, tissue consumption, sulfide speciation, and distance from the delivery source. Consequently, early implant studies should characterize concentration–time and spatial exposure profiles alongside cytotoxicity and biological efficacy rather than assume that concentrations reported in other experimental systems will translate directly to neural tissue.

Implementing any of these strategies requires careful integration with device materials and function. H₂S delivery components must not compromise electrode impedance, signal fidelity, or long-term structural stability. At the same time, the therapeutic objective is temporally constrained: the proposed intervention is intended primarily for the acute-to-subacute post-implantation period, when oxidative stress, inflammatory signaling, and tissue remodeling are especially dynamic. The optimal exposure duration remains unknown and should therefore be treated as an experimental variable rather than a predefined treatment window. Chronic neural implants impose stricter constraints than most drug-eluting platforms, particularly with respect to electrical performance and long-term stability. These limitations suggest that passive release strategies may be insufficient for achieving precise temporal and dose control at the neural interface.

These constraints motivate consideration of delivery paradigms that provide greater temporal precision and control than passive release. One such approach is electro-activated H₂S precursor delivery, in which electrically triggered reactions could generate brief, localized pulses of bioavailable sulfide. This approach aligns with implantable neurotechnology by enabling temporal control, dose-per-pulse regulation, and integration with existing stimulation paradigms. A key testable engineering requirement would be establishment of a reproducible relationship between electrical input and resulting local sulfide exposure, potentially enabling tunable or closed-loop modulation of the peri-implant environment. However, significant challenges remain, including identification of suitable precursor chemistries, quantification of electrochemical conversion efficiency, electrode stability, and avoidance of off-target electrochemical reactions.

## 6 Discussion

### 6.1 Testable predictions

The central hypothesis that localized, time-limited H₂S delivery can modulate the neural implant-tissue interface leads to several experimentally testable predictions:

1. *Reduction in peri-implant inflammation and oxidative stress*: Neural implants with controlled H₂S delivery are expected to exhibit reduced gliosis and inflammatory markers (e.g., GFAP, Iba1, pro-inflammatory cytokines) and lower oxidative stress relative to controls.
2. *Improved neuronal preservation at the implant interface*: H₂S-modulated implants are expected to show increased neuronal density and viability in the peri-implant region (e.g., NeuN-positive cells), indicating reduced implant-induced neurodegeneration.
3. *Biphasic dose–response relationship*: H₂S delivery is expected to exhibit a dose-dependent, non-linear profile, with low (nM-low μM) concentrations producing protective effects and higher or sustained concentrations leading to diminished benefit or toxicity. This reflects the dual role of H₂S in mitochondrial modulation and hypoxia-mimetic signaling.
4. *Temporal dependence of therapeutic efficacy*: Transient H₂S exposure during the acute-to-subacute post-implantation period is expected to produce greater improvements than continuous or delayed exposure, consistent with time-limited activation of adaptive pathways.
5. *Localized activation of hypoxia-responsive signaling without systemic effects*: H₂S delivery confined to the implant interface is expected to induce localized HIF-1α stabilization and downstream signaling (e.g., VEGF, Nrf2) without systemic hypoxia or widespread metabolic suppression.
6. *Improved electrophysiological stability of neural recordings or stimulation*: Devices incorporating effective H₂S modulation are expected to exhibit improved long-term performance, including more stable impedance, higher signal-to-noise ratio, and increased unit yield.
7. *Enhanced control with temporally precise delivery paradigms*: Temporally precise delivery strategies (e.g., electro-activated precursor systems) are expected to produce more consistent and tunable outcomes than passive diffusion-based methods.

### 6.2 Experimental framework

To evaluate these predictions, we outline an illustrative experimental framework based on standard neural interface benchmarks. The objective is to determine whether localized, time-limited H₂S exposure modulates the foreign body response and improves implant-tissue integration.

First, *in vitro* systems can be used to characterize H₂S release kinetics and verify biological activity in neuronal and glial cultures, ensuring that delivered concentrations engage antioxidant or anti-inflammatory pathways without inducing cytotoxicity. Release characterization should include concentration–time profiles, spatial diffusion from the delivery platform, and identification of exposure levels associated with loss of cell viability. Readouts include reactive oxygen species (ROS) assays, pro-inflammatory cytokine measurements (e.g., TNF-α, IL-1β), and markers of neuronal viability or neurite outgrowth. Standard analytical tools, including sulfide sensors or fluorescent probes, may be used to quantify local H₂S exposure (Peng et al., 2011). Testing multiple concentrations and exposure durations would allow the expected dose-dependent transition from ineffective to protective and ultimately toxic exposure to be defined. These studies assess whether delivery strategies operate within a biologically active, non-toxic range.

Second, *in vivo* rodent implantation models can compare H₂S-modulated devices with conventional implants to assess effects on device performance and tissue response over time. Electrophysiological metrics such as signal-to-noise ratio, unit yield, and electrochemical impedance spectroscopy (EIS) can be used to quantify functional device performance. In particular, experiments should assess whether transient H₂S exposure during the acute-to-subacute post-implantation period is sufficient to produce sustained improvements in tissue response and device performance. Typical endpoints include histological measures of gliosis and neuronal preservation, including GFAP and Iba1 labeling for glial activation and NeuN for neuronal density, ideally quantified as a function of distance from the implant, alongside monitoring for systemic effects, enabling estimation of effective dose ranges and exposure windows. Control conditions (e.g., non-H₂S-releasing coatings or sham treatments) are required to isolate H₂S-specific effects. Because H₂S is also a neuromodulator, changes in electrophysiological performance should be interpreted alongside the possibility of direct effects on neuronal excitability or synaptic transmission, rather than being attributed solely to improved tissue integration. Where technically feasible, measurements of local sulfide concentration should be paired with these outcomes to relate biological and functional effects directly to peri-implant exposure.

Finally, translational considerations include long-term coating stability, reproducible release kinetics, and compatibility with chronic implantation. Additional factors include characterization of donor degradation products, potential accumulation or off-target distribution of sulfide species, and regulatory classification as a combination product, which would require independent validation of both device performance and controlled chemical release. Together, these stages determine whether the proposed hypoxia-mimetic mechanism translates into measurable improvements in neural interface performance. The hypothesis would be supported if an appropriately bounded H₂S exposure improves both tissue-level and functional outcomes relative to controls, and weakened if such benefits are absent, transient, or achievable only at exposures associated with toxicity.

### 6.3 Future outlook and challenges

Several challenges remain before H₂S-based implant therapies become feasible. Foremost, safety and dosing must be tightly controlled: H₂S exhibits a bell-shaped dose–response, with protective effects confined to a narrow therapeutic window and toxicity emerging at supraphysiological levels. In the context of hypoxia-mimetic signaling, insufficient activation may fail to engage protective pathways, while excessive or prolonged exposure may induce metabolic suppression or maladaptive responses. Although systemic and localized exposure differ, both underscore the need for precise dose regulation.

A second challenge concerns treatment duration. H₂S signaling may be required only during the acute-to-subacute post-implantation period (days to weeks), when inflammatory and oxidative responses are most dynamic, rather than throughout the implant’s lifetime. The optimal temporal profile remains unknown and may depend on device type, implantation site, and patient-specific factors. This temporal constraint may reduce the need for long-term delivery stability, instead favoring systems optimized for short-duration, high-control release during the initial post-implantation window. Determining whether transient engagement of hypoxia-linked protective programs is sufficient will inform whether finite donors or replenishable systems are necessary.

Third, from a translational perspective, H₂S-releasing neural implants would almost certainly be classified as Combination Products (device plus drug/biologic) by regulatory agencies such as the U. S. Food and Drug Administration. Key considerations include demonstrating controlled and reproducible release kinetics, long-term stability of the donor within the coating or reservoir, absence of toxic leachables, and independent safety profiles for both the device and the bioactive component. Variability in local tissue responses and sulfide metabolism may further complicate dosing standardization across patients. Addressing these requirements early will be critical for moving H₂S-based neurotechnologies beyond proof-of-concept studies toward clinical translation.

Finally, reliable measurement of local H₂S concentrations remains a key translational challenge. Fluorescent probes enable sensitive detection and imaging of sulfide species, but can suffer from photobleaching, limited penetration depth, and interference from related sulfur-containing molecules (Peng et al., 2011; Lippert et al., 2011; Liu et al., 2011). Continuous permeability-based detection methods provide improved chemical specificity but remain difficult to adapt to implant-adjacent, spatially resolved monitoring (Faccenda et al., 2012). Amperometric and other electrochemical sensors offer real-time detection and may be more compatible with implant-integrated or implant-adjacent monitoring (Jeroschewski et al., 1996; MalekAlaie et al., 2015; Zhao et al., 2018). However, electrochemical approaches face challenges related to drift, biofouling, selectivity against other redox-active species, and calibration in complex neural tissue environments (Jeroschewski et al., 1996; MalekAlaie et al., 2015; Zhao et al., 2018).

Accurately resolving peri-implant gradients is further complicated by the coexistence of free H₂S/HS^−^, polysulfides, and bound sulfane sulfur pools. This chemical complexity introduces ambiguity in interpreting measured signals and correlating concentrations with biological effects. These limitations currently constrain closed-loop implementations, which would require robust long-term sensing, control algorithms, and safety gating to be clinically viable.

More broadly, this framework suggests that controlled modulation of local biochemical environments may represent a generalizable strategy for improving the performance of implantable devices beyond neural interfaces.

### 6.4 Conclusion

Hydrogen sulfide is an endogenous neuromodulator with cytoprotective, antioxidant, and anti-inflammatory functions in the nervous system. Its ability to engage hypoxia-like adaptive pathways and modulate oxidative stress and inflammation supports its potential to address the hostile tissue response to chronic neural implants. This Hypothesis and Theory article advances the testable hypothesis that localized, temporally bounded H₂S delivery can function as a controlled hypoxia mimetic at the implant–tissue interface, reducing reactive gliosis, preserving peri-implant neurons, and ultimately improving electrophysiological stability.

This framework integrates mechanistic evidence, delivery constraints, and interface-specific considerations to identify the central experimental question: whether the hypoxia-mimetic effects of H₂S can be reproduced safely and consistently at a neural interface. Addressing this question will require evaluation across defined dose ranges, temporal exposure profiles, and delivery paradigms compatible with chronic implantation. More broadly, the hypothesis reflects a shift from treating the neural implant as a passive material interface toward actively controlling its biochemical microenvironment.

Important limitations of this hypothesis include the absence of implant-specific H₂S dose–response data, uncertainty regarding local sulfide speciation and diffusion within neural tissue, and the lack of chronic *in vivo* studies demonstrating that H₂S-mediated tissue protection translates into improved electrophysiological performance. These uncertainties also make the central hypothesis directly falsifiable: temporally bounded local H₂S delivery should improve defined histological and functional measures of chronic neural-interface integration relative to appropriate controls, whereas ineffective or excessive exposure should fail to provide these benefits. If validated, this approach could establish H₂S-mediated biochemical modulation as a new strategy for improving the performance and longevity of implantable neurotechnologies.

## Data Availability

The original contributions presented in the study are included in the article/supplementary material, further inquiries can be directed to the corresponding author.
